# Remifentanil inhibits the inflammatory response of BV2 microglia and protects PC12 cells from damage caused by microglia activation

**DOI:** 10.1080/21655979.2022.2080421

**Published:** 2022-06-20

**Authors:** Yankui Huang, Qingxiang Cai, Huihui Liu, Yong Wang, Wuhua Ma

**Affiliations:** Department of Anesthesiology, The First Affiliated Hospital of Guangzhou University of Chinese Medicine, Guangzhou, P.R. China

**Keywords:** Neuroinflammation, Remifentanil, BV2 microglia, PC12 cells, lipopolysaccharide

## Abstract

Microglia acts as a critical player in neuroinflammation and neuronal injury. Remifentanil (Rem) has been reported to exert anti-inflammatory activity in several types of diseases. However, the role of Rem in microglia-mediated neuroinflammation is unclear. The present study was designed to investigate the effects of Rem against lipopolysaccharide (LPS)-activated BV2 microglial and PC12 cell induced by activated BV2 microglia. Cell proliferative ability was assessed with cell counting kit-8 assay and cellular morphology was observed. ELISA assay was used to measure the expressions of PGE2 and inflammatory factors. The contents of p-NF-KB p65, p-IKKα/β, and COX2 were evaluated with the aid of western blot. The levels of NO and iNOS were assessed with Griess assay, qRT-PCR, and western blot. In addition, Tunel assay and western blot were performed to assess cell apoptosis. The data revealed that Rem alleviated BV2 microglial morphological injury induced by LPS. Furthermore, Rem suppressed inflammatory releases, iNOS, NO and PGE2 stimulated by LPS in activated BV2 cells. Moreover, Rem suppressed PC12 cell injury, the generations of inflammatory factors and cell apoptosis triggered by inflammatory mediators secreted from activated BV2 cells. These results suggest that Rem exhibited anti-neuroinflammatory activity in protecting PC12 cells against injury derived from LPS-stimulated BV2 microglia.

## Graphical abstract

**Figure:**
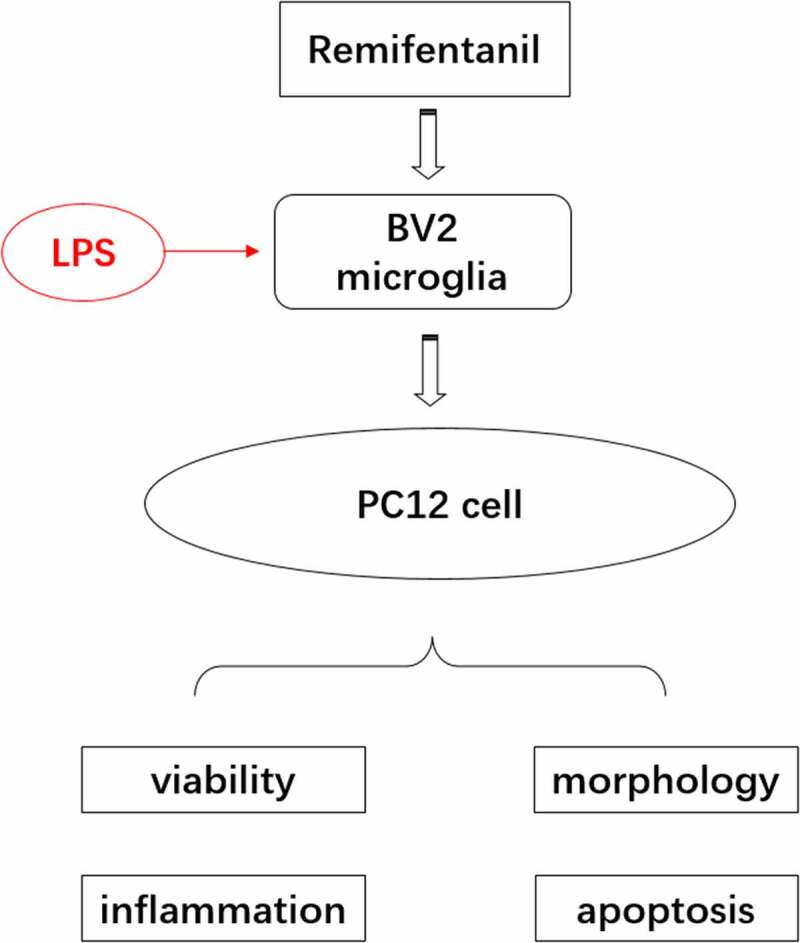

## Highlights

- Rem inhibits the release of LPS-induced inflammatory factors in BV2 cells.
- Rem reduces activated microglia-induced damage to cytoactive of PC12 cells.
- Rem attenuates levels of activated microglia-induced inflammatory factors and apoptosis in PC12 cells.

## Introduction

Neuroinflammation is an important factor of many central nervous system (CNS) diseases including Alzheimer, Parkinson, and other neurodegenerative conditions [1–3]. The process of neuroinflammation is a complex brain injury response involved in the activation of glia, release of inflammatory mediators and production of ROS [4,5]. Being the smallest glial cells, microglia are judged to be the resident macrophages of CNS [6]. ‘Resting’ microglia morphologically transform into ‘activated microglia’ by lipopolysaccharide (LPS), leading to neuronal injury via facilitating the generations of cytotoxic molecules, such as the pro-inflammatory cytokines TNF-α, IL-6, and IL-1β, as well as chemotactic cytokines [7,8]. Hence, inhibition of pro-inflammatory mediators and cytokines induced by microglial activation is deemed to be a prospective effective strategy for the prevention and treatment of central nervous system diseases [9].

Remifentanil (Rem) is a potent μ-opioid receptor agonist, mainly used to induce general anesthesia and maintain analgesia during general anesthesia clinically [10–12]. It has been reported that Rem plays anti-inflammation role in many diseases associated with inflammation [13]. For instance, Rem suppressed the production of IL-6 and iNOS in septic mice [14]. In addition, Rem ameliorated LPS-induced acute lung injury by the inhibition of proinflammatory cytokine via downregulating the NF-κB pathway [15]. However, the functional role and the mechanism behind this protective effect of remifentanil in neuroinflammation are not distinct yet.

In the present study, we aimed to explore the protective effects of Rem on inflammation response from LPS-activated microglia and underlying mechanism in PC12 dopaminergic neurons.

## Materials and methods

### Cell culture and treatment

BV2 microglial cells were bought from the National Platform of Experimental Cell Resources for Sci-Tech, Cell Resource Center (Beijing, China). PC12 cells were obtained from the Institute of Basic Medical Sciences of the China Science Academy. DMEM decorating with 10% FBS, 100 U/ml penicillin, and 100 μg/ml streptomycin was utilized to cultivate the cells. To activate the microglia, LPS (1 μg/mL) was employed to administrate BV2 cells for 24 h. After the simulation with 40 ng/ml of nerve growth factor (NGF) in DMEM containing 1% HS and 0.5% FBS for 3–6 days, the differentiation of PC12 cells into neuron-like cells was accomplished. 20 ng/ml of PMA was added into BV2 cells FOR 24 h to act as NF-κB activator.

To explore the effects of neurotoxic factors released by BV2 cells on PC12 cells, the pretreatment and stimulation of BV2 cells were carried out with varying doses of remifentanil (Rem; Renfu, China) and LPS, separately. Following the centrifugation of collected cell culture media was operated, the supernatant was utilized to be BV2 microglia-conditioned medium (CM). PC12 cells that inoculated into 6-well plates were administrated with CM for 24 h.

### Cell counting kit-8 (CCK-8) assay

BV2 cells that inoculated into 96-well plates were administrated with 0, 0.5, 1, and 2 μM Rem for 30 min before the addition or absence of LPS for another 24 h. The cell viability of PC12 in response to neurotoxic factors released by BV2 cells was detected by CCK-8 assay as well. PC12 that inoculated into 96-well plates were cultured with the supernatants of BV2 cells with different treatments for 24 h. Thereafter, each well was added with 10 μL of WST-8 in serum-free DMEM and incubated for 3 h. The OD value was decided by a microplate reader in the premise of λ = 450 nm [16].

### Morphological observation

Varying doses of Rem (0, 0.5, 1, and 2 μM) were applied to administer BV2 cells that were plated in 6-well plates. After 48 h stimulation with 1 μg/mL LPS, cellular morphology of the BV2 cells in each group were observed and photographed with the EOS Utility Software (Canon, USA). In addition, morphological change of PC12 cells was photographed after being treated with CM using EOS Utility Software.

### ELISA assay

The levels of inflammatory mediator PGE2 and pro-inflammatory cytokines including TNF-α and IL-6 were detected by using ELISA assay. Briefly, Rem was adopted to administer BV2 cells for 0.5 h prior to the induction with LPS for 24 h. Then, PC12 cells was added with CM from BV2 cells and cultured for 24 h. The culture media of PC12 cells was collected and the supernatant was obtained after centrifugation. The concentrations of inflammatory mediators in CM from BV2 cells and the supernatant from PC12 cells were assayed by ELISA kits (Beyotime Institute of Biotechnology, Jiangsu, China).

### Quantitative real-time polymerase chain reaction (qRT-PCR)

The isolation of total RNAs from BV2 cells treated with or without Rem in the presence or absence of LPS was carried out by Trizol reagent (Invitrogen, Carlsbad, CA, USA). Reverse transcription (RT) of first-strand cDNAs was performed by using PrimeScript RT Master Mix (Perfect Real Time; Takara Bio, Inc., Tokyo, Japan). With the aid of SYBR Premix ExTaq kit (Takara Bio, Inc.), PCR reactions were operated on ABI PRISM 7900 Real-Time system (Applied Biosystems). The calculation of relative expression of target genes was performed by relative quantification (2^−ΔΔCt^) method [17].

### Western blot assay

The administration of BV2 cells with 0.5, 1, and 2 μM Rem was carried out, before which was the induction with LPS for 24 h. PC12 cells were subjected to the CM. The total proteins were extracted from treated BV2 cells and PC12 cells using RIPA buffer (Bio-Rad, CA, USA). Following the exposure to SDS-PAGE, the transferring of proteins was operated onto PVDF membranes. Subsequently, the overnight incubation with primary antibodies (anti-p-NF-κB p65, p-IKKα/β, NF-κB p65, IKKα/β, COX2, iNOS, Bax, Bcl-2, cleaved caspase 3, cleaved PARP, anti-GAPDH) was accomplished at 4°C, following which was the cultivation with HRP-conjugated secondary antibody. The antibody-labeled proteins were detected by Imagequant LAS 4000 (GE Healthcare) system with the Western Lighting Plus-ECL (PerkinElmer, 203–17,201). The density of the band was tracked by the Image J software (NIH, Bethesda, MD, USA).

### Griess assay

With the adoption of Griess assay, the generation of NO was assayed [18]. BV2 cells (5 × 10^5^ cells/mL) that were inoculated into 24-well plates were pretreated with 0.5, 1, and 2 μM Rem for 30 min before induction with LPS for 24 h. Same volume of the Griess reagent was applied to mix with collected culture supernatants. NO concentration was decided when the absorbance at 540 nm with a microplate reader.

### TdT-mediated dUTP nick-end labeling (TUNEL) assay

PC12 cell apoptosis was detected by TUNEL assays with a TUNEL detection kit according to manufacturer’s instruction (Roche, Germany). In brief, the administration and fixation of PC12 cells with CM and 4% paraformaldehyde were carried out. Following the cultivation with proteinase K for 15 min in 37°C, cells were placed in 3% H_2_O_2_. After the rinse with PBS for several times, cells were treated by TUNEL detection kit and images were captured by a fluorescence microscopy (Olympus BX53, Japan). Positive cells were counted, and at least 10 randomly chosen fields for each sample were examined per section.

### Statistical analysis

All data that reported as mean ± SD were analyzed with SPSS (Version 21.0) as well as GraphPad Prism (version 5.0, CA, USA). All assays were independently repeated three times. One-way ANOVA followed by Bonferroni post hoc comparison tests was applied to show comparisons among multiple groups. P less than 0.05 were supposed to show statistical significance.

## Results

In this study, we studied the effects of Rem in inflammatory response in LPS-stimulated BV2 microglia and PC12 cell injury. The results revealed that Rem restrained BV2 microglial morphological injury. In addition, Rem inhibited the inflammatory releases, and production of iNOS, NO and PGE2 stimulated by LPS in activated BV2 cells. Moreover, Rem suppressed PC12 cell injury, the generations of inflammatory factors and cell apoptosis triggered by inflammatory mediators secreted from activated BV2 cells.

### Effects of Rem on cell viability of untreated or LPS-activated BV2 cells

**Figure 1.**
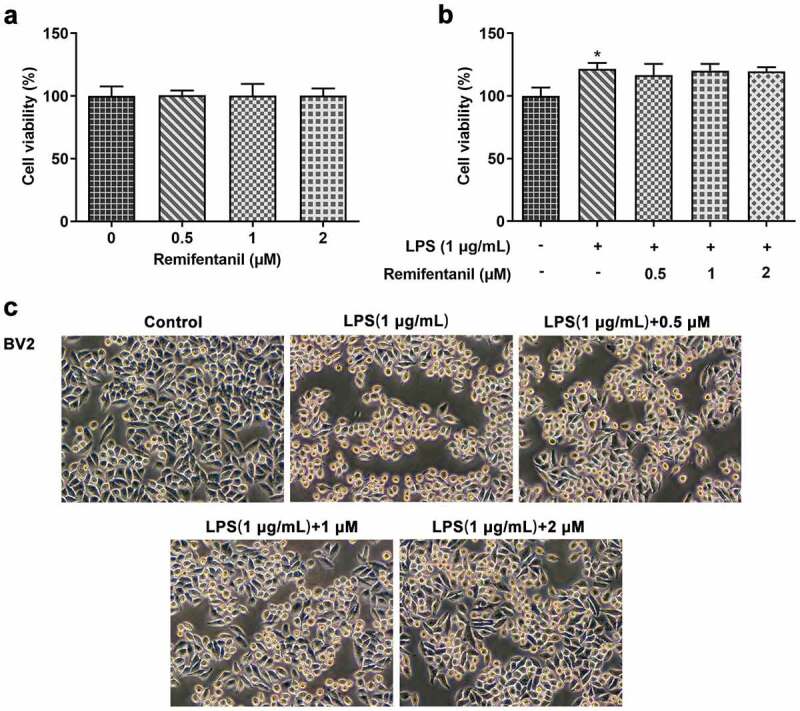
Effects of Remifentanil (Rem) on cell viability and morphology in LPS-induced BV2 cells. a, Cell viability of BV2 cells treated with 0–2 μM Rem. b, Cell viability of untreated or LPS-induced BV2 cells in the presence of 0–2 μM Rem. c, Cell morphology of untreated or LPS-induced BV2 cells with or without 0–2 μM Rem. Results are the mean ± SD. *P < 0.05 versus control.

First, we examined the cytotoxicity of Rem on BV2 treated with or without LPS by CCK-8 assays. As shown in Figure 1(a), cell viability of control BV2 cells was not affected by Rem within 2 μM. Then, we observed that the stimulation of LPS elevated the cytoactive of BV2 cells, while Rem didn’t impact the cell viability in BV2 cells induced by 1 μg/ml LPS (Figure 1(b)). In addition, larger round cell body and less retracted branches were observed in LPS-stimulated cells compared with those in control cells. However, Rem treatment led to the ameliorated morphology of BV2 cells after induction of LPS (Figure 1(c)).

### Effects of Rem on release of LPS-induced inflammatory factors in BV2 cells

**Figure 2.**
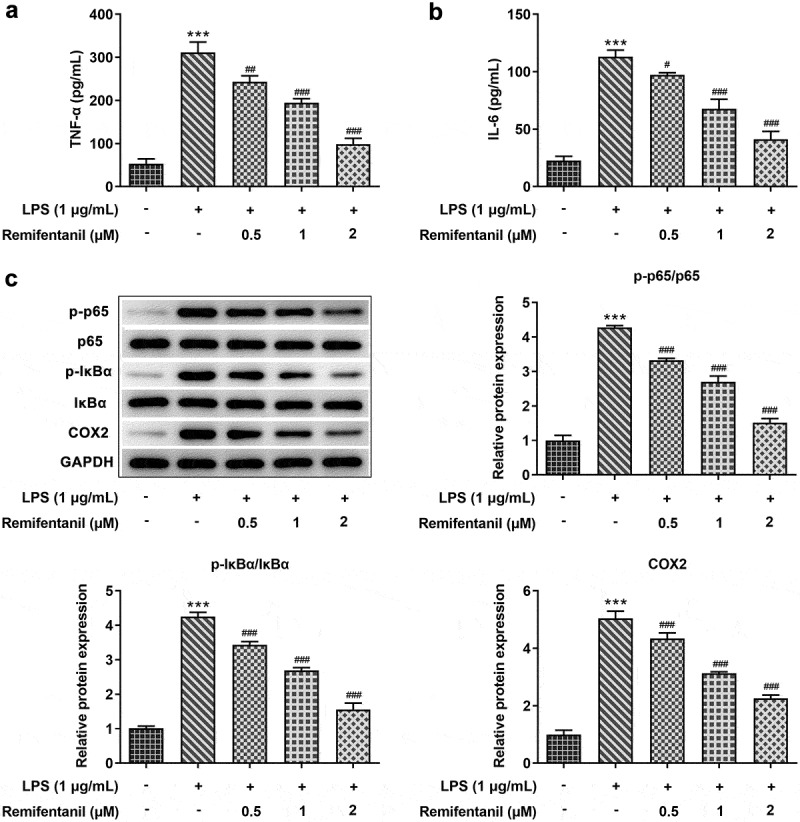
Effects of Rem on production of inflammatory factors in LPS-induced BV2 cells. Levels of TNF-α (a) and IL-6 (b) in BV2 cells treated with or without Rem were detected by ELISA assay. C, Protein levels of NF-κB pathway and COX2 in BV2 cells treated with or without Rem were measured by western blot assay. Results are the mean ± SD. ***P < 0.001 versus control. ^#^P < 0.05, ^##^P < 0.01, ^###^P < 0.001 versus LPS group.

To explore the influences of Rem on neuroinflammation, the releases of inflammatory factors in BV2 cells were tracked. As Figure 2(a, b) depicted, LPS induction hugely enhanced the levels of TNF-α and IL-6 in BV2 cells when compared with those in control cells, while pretreatment with Rem was found to obviously reduce the generations of these cytokines. Moreover, results from western blot assay revealed that in LPS-treated cells, in contrast with that in cells without any treatment, protein contents of p-NF-κB p65, p-IKKα/β, and COX2 were greatly elevated. Nonetheless, Rem treatment efficiently reversed the activation of LPS on inflammation-associated proteins (Figure 2(c)), suggesting that Rem inhibited secretions of LPS-induced pro-inflammatory cytokines in BV2 cells.

### Effects of Rem on LPS-induced iNOS expression and production of NO and PGE_2_ in BV2 Cells

**Figure 3.**
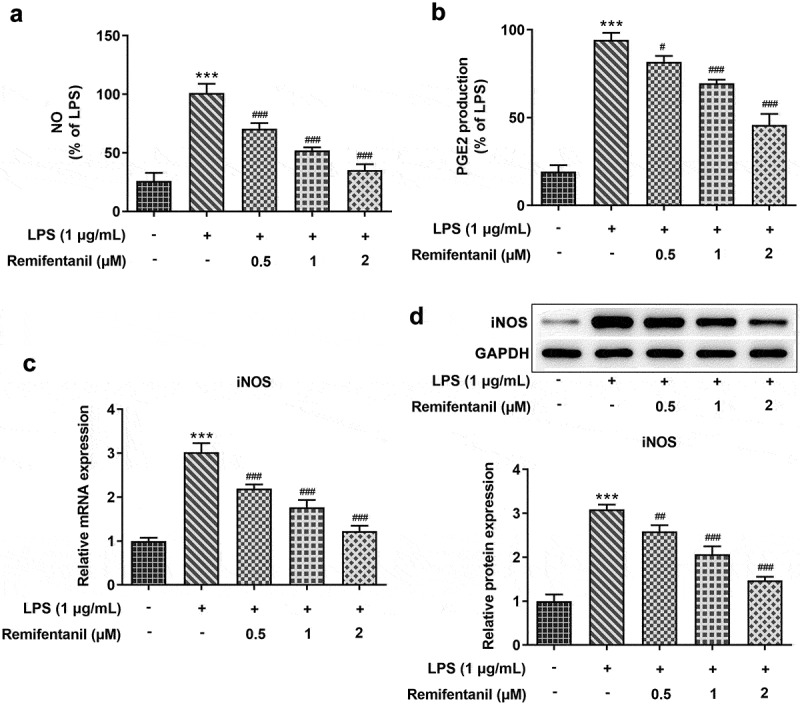
Effects of Rem on production of NO, PGE2 and iNOS expression in LPS-stimulated BV2 Cells. The amount of NO (a) and PGE2 (b) were determined by Griess’s reagent assay and ELISA assay, respectively. mRNA level (c) and protein expression (d) of iNOS was identified by qRT-PCR and western blot assay, respectively. Results are the mean ± SD. ***P < 0.001 versus control. ^#^P < 0.05, ^##^P < 0.01, ^###^P < 0.001 versus LPS group.

To further investigate the effects of Rem on inflammatory injury in neurodegenerative diseases, BV2 cells were administered with 0.5, 1, or 2 μM of Rem for 0.5 h, then induced with 1 μg/ml of LPS for 24 h. The results demonstrated that LPS dramatically enhanced the production of NO and PGE_2_ while pretreatment of Rem suppressed the levels of NO and PGE_2_ in a concentration-dependent manner in BV2 Cells (Figure 3(a,b)). Consistent with this data, mRNA and western blot assay showed that iNOS level was stimulated by 1 μg/ml of LPS, whereas Rem exerted the opposite effect on the mRNA and protein expression of iNOS in LPS-stimulated microglial cells (Figure 3(c, d)). The results indicate that Rem repressed LPS-stimulated production of NO and PGE_2_ and suppressed iNOS expression in BV2 cells.

### Rem inhibits the release of inflammatory factors, iNOS, NO, and PGE2 through NF-κB signaling in BV2 Cells

**Figure 4.**
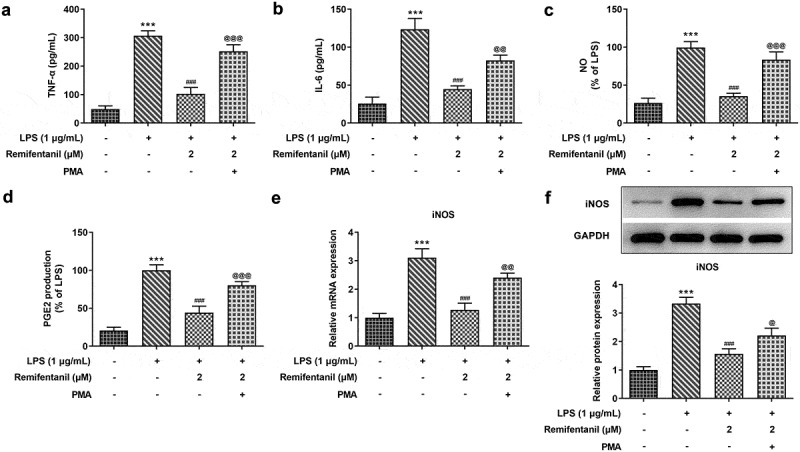
Effects of PMA on production of inflammatory factors, NO, PGE2 and iNOS expression in LPS-stimulated BV2 Cells with Rem treatment. Levels of TNF-α (a) and IL-6 (b) in BV2 cells treated with or without PMA were detected by ELISA assay. The amount of NO (c) and PGE2 (d) were determined by Griess’s reagent assay and ELISA assay, respectively. mRNA level (e) and protein expression (f) of iNOS was identified by qRT-PCR and western blot assay, respectively. Results are the mean ± SD. ***P < 0.001 versus control. ^###^P < 0.001 versus LPS group. ^@@^P < 0.01, ^@@@^P < 0.001 versus LPS+Rem (2 μM) group.

As shown in Figure 4(a, b), Rem significantly reduced the secretion of TNF-α and IL-6 in BV2 cells but PMA undermined the effects of Rem on TNF-α and IL-6. In addition, Rem dramatically suppressed the production of NO and PGE2, while treatment of PMA rehabilitated the levels of NO and PGE2 in BV2 Cells (Figure 4(c, d)). Moreover, data from PCR and western blot assay showed that iNOS level was inhibited by Rem, while PMA enhanced the expression of iNOS in Rem-treated microglial cells stimulated by LPS (Figure 4(e, f)). The results indicate that NF-κB signaling was associated with the function of Rem on the release of inflammatory factors, iNOS, NO, and PGE2 in BV2 Cells.

### Rem reduces activated microglia-induced damage to cytoactive of PC12 cells

**Figure 5.**
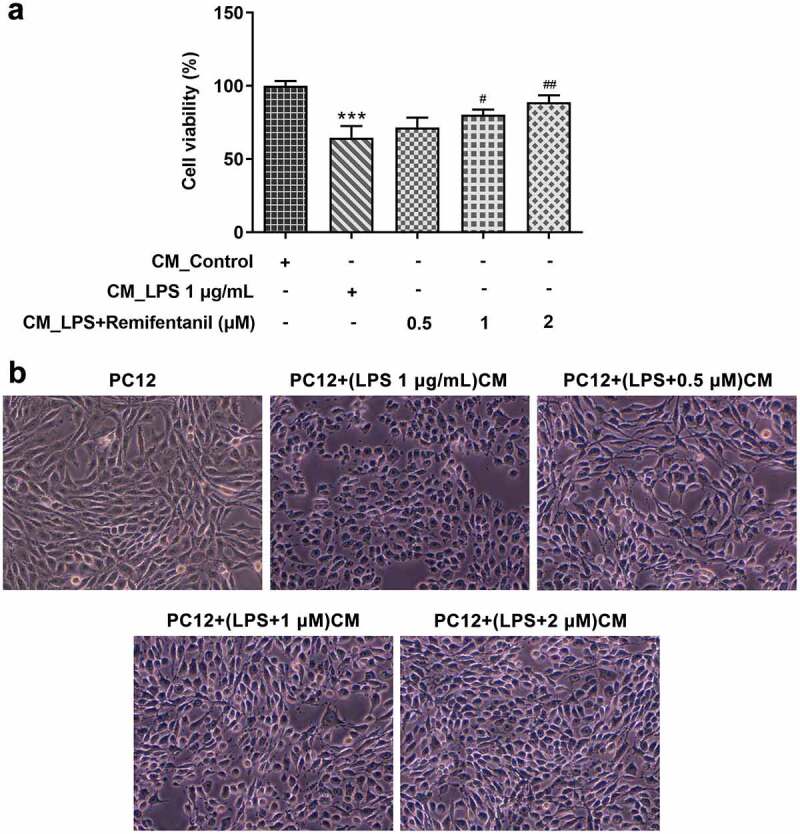
Effects of Rem on cell viability and morphology of PC12 neurons induced by conditioned medium from LPS-stimulated BV2 cells. BV2 cells were pretreated with Rem (0.5, 1 and 2 μM) for 30 min and stimulated with 1 μg/mL LPS for 24 h. Conditioned medium (CM) from BV2 cells was collected and PC12 cells were cultured in conditioned medium for 24 h. a, Cell viability of PC12 cells was detected by CCK-8 assay. b, Cell morphology of PC12 cells was observed by a light microscope. Results are the mean ± SD. ***P < 0.001 versus CM_control group. ^#^P < 0.05, ^##^P < 0.01 versus CM_LPS group.

To examine whether Rem has protective effects on nerve cells under inflammatory conditions induced by microglial activation, PC12 were cultured in conditioned media from BV2 cell culture. As Figure 5(a) demonstrated, compared with PC12 cells plated into normal medium, PC12 cells treated with CM from LPS-activated BV2 cells exhibited lower cell viability. However, Rem treatment reduced the inhibitory effects of CM from LPS-activated BV2 cells on the viability of PC12 cells. Similarly, the morphology of PC12 cells exposed to CM from LPS-stimulated BV2 cells showed impaired cell morphology with smaller cell body and less neurite, while CM from Rem-treated BV2 cells alleviated the damage of LPS-stimulated BV2 cells to cell morphology of PC12 cells (Figure 5(b)). Thus, Rem protected the cytoactive and morphology of PC12 exposed to CM from activated microglia.

### Rem attenuates levels of activated microglia-induced inflammatory factors and apoptosis in PC12 cells

**Figure 6.**
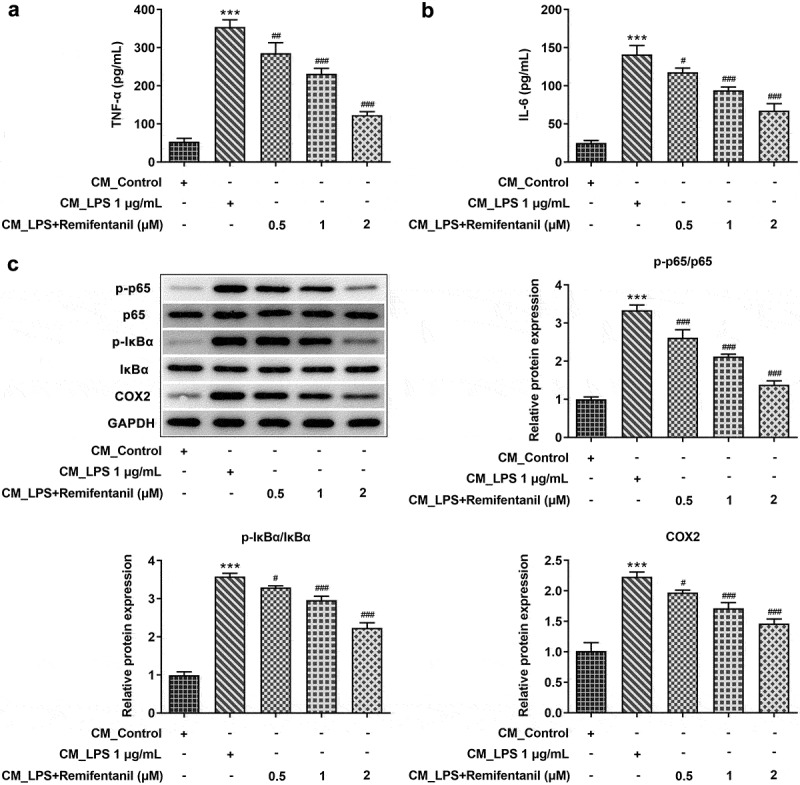
Effects of Rem on production of inflammatory factors in PC12 neurons induced by conditioned medium from LPS-stimulated BV2 cells. BV2 cells were pretreated with Rem (0.5, 1 and 2 μM) for 30 min and stimulated with 1 μg/mL LPS for 24 h. Conditioned medium (CM) from BV2 cells was collected and PC12 cells were cultured in conditioned medium for 24 h. Levels of TNF-α (a) and IL-6 (b) in PC12 cells were detected by ELISA assay. c, Protein levels of NF-κB pathway and COX2 in PC12 cells were measured by western blot assay. Results are the mean ± SD. ***P < 0.001 versus CM_control group. ^#^P < 0.05, ^##^P < 0.01, ^###^P < 0.001 versus CM_LPS group.

**Figure 7.**
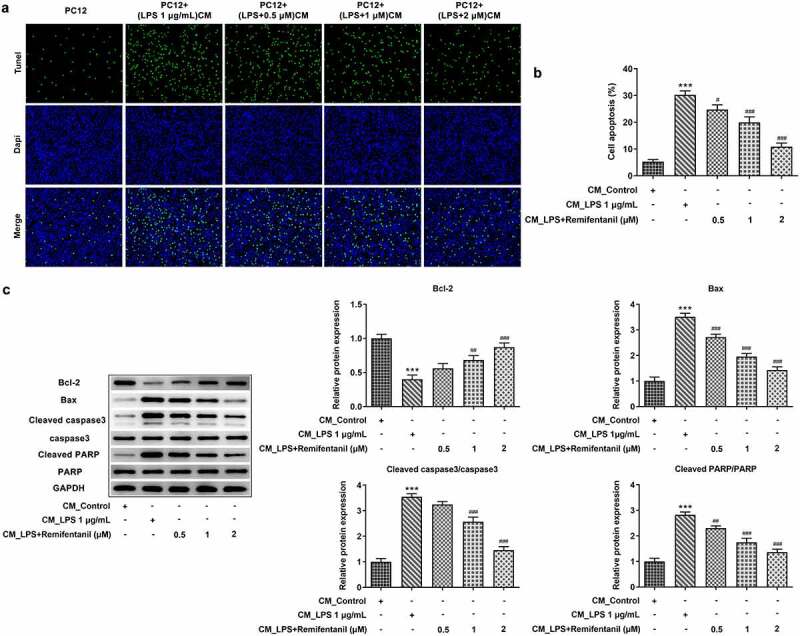
Effects of Rem on the apoptosis of PC12 neurons induced by conditioned medium from LPS-stimulated BV2 cells. BV2 cells were pretreated with Rem (0.5, 1 and 2 μM) for 30 min and stimulated with 1 μg/mL LPS for 24 h. Conditioned medium (CM) from BV2 cells was collected and PC12 cells were cultured in conditioned medium for 24 h. a and b, Cell apoptosis of PC12 cells was assessed by TUNEL assay. c, Western blot assay was performed to measure the levels of apoptosis-related proteins in PC12 cells. Results are the mean ± SD. ***P < 0.001 versus CM_control group. ^##^P < 0.01, ^###^P < 0.001 versus CM_LPS group.

Finally, we explored the role of Rem in inflammatory responses and cell apoptosis of PC12 cells exposed to CM from LPS-stimulated microglia. As Figure 6 presented, the protein contents of TNF-α, IL-6, p-p65, p-IKKα/β, and COX2 were all increased in PC12 cells treated with CM from LPS-activated BV2 cells, by contrast with cells fostered in normal medium. However, pretreatment of Rem counteracted the secretions of those proinflammatory mediators in a dose-dependent manner in PC12 cells with CM of LPS-treated BV2 cells. Additionally, activated microglia-induced neuronal cell apoptosis was measured by TUNEL assay and western blot analysis. As Figure 7(a, b) illustrated, the apoptosis rate of PC12 cells was notably increased after treatment of CM from LPS-stimulated BV2 cells, while Rem treatment in BV2 cells specifically reduced the apoptosis rate of PC12 cells. Consistently, an obvious decrease in Bcl-2 level and increases in levels of Bax, cleaved casapse3, and cleaved PARP were observed in PC12 cells exposed to CM from Rem-treated BV2 cells (Figure 7(c)). These data suggest that Rem reversed releases of inflammatory factors and apoptosis induced by LPS-stimulated microglia in PC12 cells.

## Discussion

In this study, we demonstrated that Rem could suppress LPS-stimulated neuroinflammation and cell injury in microglia. Furthermore, we found that Rem alleviated PC12 cell injury and apoptosis induced by conditioned medium from LPS-activated BV2 microglia.

It is well known that LPS could function as a stimulator that results in changes of morphology, physiology, and pathology in cells [19–21]. When microglia was exposed to LPS administration, it is compelled to turn into an activated state, showing the features of amoeboid morphology with enlargement of cell bodies and retracted processes [22]. In the current study, we used 1 μg/mL LPS to stimulate microglia BV2 cells and the results revealed the activation of BV2 cells by increased cell viability and typical morphological changes. Then, 0–2 μM of Rem was added into LPS-activated BV2 cells, and we found that, the cellular morphology changes at activated condition by LPS were recovered as the increased concentration of Rem, indicating the plays anti-inflammation role of Rem in BV2 cells induced by LPS.

Potential neurotoxic molecules, including inflammatory mediators were released from activated microglial, in response to CNS damages or immunostimulation [23,24]. Evidence has suggested that NO and PGE_2_ are pivotal inflammatory mediators in the pathogenesis of many inflammation-related neurodegenerative diseases [25]. NO and PGE_2_ are derived from inducible isoforms of iNOS and COX-2 enzymes, respectively [26]. We found that LPS treatment significantly stimulated the production of NO and PGE2, but Rem prevented LPS-induced upregulation in NO and PGE2 in BV2 cells in a dose-dependent manner. Correspondingly, Rem led to a decrease of iNOS and COX-2 production, which was consistent with previous results [27].

Excessive pro-inflammatory cytokines have been observed in numerous neurodegenerative diseases [28]. TNF-α and IL-6, the key pro-inflammatory cytokines secreted by activated microglia, participate in initiating and promoting the cytokine cascade with glial cell reactions during inflammatory response in neuroinflammation [29]. Our results demonstrated that pretreatment with Rem markedly reduced the production of pro-inflammatory mediator including TNF-α and IL-6 in LPS-induced BV2 microglia. Microglial-neuronal interactions are implicated in the occurrence and development of many types of neurodegenerative diseases [30,31]. Microglial activation contributes to neuronal death by directly releasing neurotoxic mediators, such as inflammatory cytokines [32]. Further research found that the conditioned medium from LPS-stimulated microglia might be harmful to PC12 cell viability and cellular morphology, and enhanced the releases of TNF-α and IL-6. Moreover, NF-κB pathway plays very important roles in neuronal inflammation [33]. Our results indicated LPS stimulation in microglial may induce the production of p-p65, p-IKKα/β, and COX2, and the conditioned medium with these proteins stimulate further generation of p-p65, p-IKKα/β, and COX2 in PC12 cells, which was in line with previous result [34]. Rem treatment reversed cytoactive and reduced the levels of pro-inflammatory cytokines in PC12 cells exposed to conditioned medium from LPS-activated microglia, providing evidences for protective effects of Rem on neure against from LPS-stimulated microglia.

Activation of microglia can cause neuronal injury and apoptosis by overproduction of inflammatory mediators. In this study, we used LPS-activated BV2 microglial conditioned medium to treat differentiated PC12 cells, which simulates the pathological conditions of neuronal damage by activated microglia in neuroinflammatory diseases [35,36]. In line with these studies, our research revealed that conditioned medium obtained from BV2 microglial without Rem treatment facilitated the apoptosis of PC12 cells, while conditioned medium of Rem-treated BV2 cells inhibited neuronal apoptosis of PC12 cells. Furthermore, we think our study has several limitations. In this study, we only chose a single timepoint for different measurements including inflammatory mediators, cell survival, and so on. Those events are likely to have different dynamics. Thus, measurement at multiple time points will be more accurate, and we will supplement them in further study. Many pathways such as caspase signaling, housekeeping pathways, and progranulin pathways have been regarded as the regulator in the activation and neurotoxicity of microglia [37–40], thus we will explore the relationship between the effects of Remifentanil on BV2 microglia and other signaling pathways.

## Conclusion

Overall, our study identified that Rem exerted anti-neuroinflammatory activity in LPS-induced activated BV2 microglial by inhibition of pro-inflammatory mediators and inflammatory cytokines. Moreover, Rem protected PC12 cells from inflammatory responses and apoptosis induced by cytotoxicity from microglial. The research provides a novel fundamental insight into how Rem protects nerve cells and gives us clues for future therapeutic strategy for neurodegenerative disorders and diseases.
